# Regulatory Effects of Chlormequat Chloride on the Yield and Chemical Composition of *Angelica sinensis* Radix

**DOI:** 10.3390/molecules29194725

**Published:** 2024-10-06

**Authors:** Honghan Qin, Juan Xu, Xiaojun Ma, Rongchang Wei, Zuliang Luo

**Affiliations:** 1 College of Pharmacy, Youjiang Medical University for Nationalities, Baise 533000, China; qinhonghanyyxb@126.com; 2 Department of Microbiology and Parasitology Faculty of Medicine, MAHSA University, Jenjarom 42610, Malaysia; 3 Biotechnology Research Institute, Guangxi Academy of Agricultural Sciences, Nanning 530007, China; 4 Institute of Medicinal Plant Development, Chinese Academy of Medical Sciences, Peking Union Medical College, Beijing 100193, China; 5 Cash Crops Research Institute, Guangxi Academy of Agricultural Sciences, Nanning 530007, China

**Keywords:** *Angelica sinensis*, chlormequat chloride, regulatory effects, chemical composition

## Abstract

Chlormequat chloride (CCC), as a commonly used plant growth regulator in the production of rhizomatous medicinal herbs, can effectively control the bolting phenomenon in *Angelica sinensis*, significantly increasing the yield of underground rhizomes (medicinal part). However, its specific effects on the intrinsic quality of *Angelica sinensis*, especially medicinal components, require further investigation. The objective of this study is to conduct a thorough examination of CCC residue and its influence on the yield and medicinal components of *Angelica sinensis*. By spraying different concentrations of CCC on *Angelica sinensis*, we systematically monitored the final yield of *Angelica sinensis* Radix (ASR) in each treatment group and the residual concentration of CCC in ASR. Using UPLC-QTOF-MS technology, we conducted an in-depth analysis of the metabolic profile of ASR. Subsequently, UFLC-MS/MS was employed to accurately quantify the changes in the content of nine key active components in ASR. The results of this study indicate that the application of CCC significantly improves the yield of ASR, with the best effect observed at 0.1 g/L, resulting in a yield increase of 24.8%. Meanwhile, the residual amount of CCC in ASR is positively correlated with the application concentration, with the residual levels as high as 7.12 mg/kg in the high-concentration treatment group. Metabolomic analysis preliminarily identified 21 chemical components in ASR, including four organic acids and 13 phthalides. It is worth noting that the quantitative analysis results indicate significant changes in active components such as butylphthalide, Z-ligustilide, and ferulic acid after the application of CCC. Specifically, high-concentration CCC significantly increased the content of butylphthalide and levistolide A, while low-concentration CCC significantly promoted the accumulation of coniferyl ferulate and senkyunolide A, accompanied by a significant decrease in Z-ligustilide and ferulic acidy. In conclusion, while CCC use can increase yield, the associated increase in residues and imbalanced composition ratios may threaten the quality and safety of ASR. Therefore, it is crucial to control the amount of CCC used rationally to balance yield enhancement and quality assurance.

## 1. Introduction

*Angelicae Sinensis* Radix (ASR), commonly known as Danggui in China, is a highly valued traditional Chinese medicinal material derived from the processed root of *Angelica sinensis* (Oliv.) Diels (Umbelliferae) [1]. This herb has been used for over 2000 years in prescriptions and formulas to enrich blood, activate blood circulation, regulate menstruation, relieve pain, and relax bowels [2,3,4]. It is also popularly known as “women ginseng” because of its numerous benefits for female health. ASR is not only widely used in Asia but also in Europe and the USA as a health food, a cosmetic ingredient, and in some animal drugs [5,6]. ASR boasts a diverse range of pharmacological properties, including anticardiovascular, neuroprotective, anticancer, antiaging, immune regulatory, and antiosteoporotic effects [7,8,9]. It is an essential component in over half of the Chinese herbal-based drugs listed in the Chinese Pharmacopoeia. Its wide applications are reflected in the saying “Ten prescriptions nine Danggui”, highlighting its prevalence in traditional Chinese medicine (TCM) [10]. ASR contains various bioactive compounds such as volatile oils, organic acids, phthalides, and polysaccharides [11,12]. Z-ligustilide and ferulic acid are the main effective components of ASR, and they are also listed as the quality control components of ASR in the 2020 edition of the Chinese Pharmacopoeia. Modern research has confirmed their efficacies in enriching blood, reducing inflammation, inhibiting platelet aggregation, preventing thrombosis, and protecting against cardiovascular and cerebral ischemia [13,14,15].

Chlormequat chloride (CCC), as a quaternary ammonium plant growth regulator (PGR), plays an important role in the agricultural field, especially in the cultivation of economic crops such as corn, wheat, tomatoes, and cotton [16,17,18]. It effectively promotes crop resistance to lodging, enhances stress tolerance, advances flowering and fruiting, and increases yield by inhibiting cell elongation without affecting cell division [19,20,21]. With the continuous expansion of medicinal herb varieties and planting areas, PGRs have begun to be frequently applied in the production of medicinal herbs. Notably, CCC is commonly applied on various rhizomatous medicinal species including *Ophiopogon japonicus*, *Achyranthes bidentata*, *Codonopsis pilosula*, *Rehmannia glutinosa*, and *Angelica sinensis*, which can rapidly promote rhizome growth and increase medicinal materials yields by 20% to 200% [22,23,24]. However, blind use and abuse in production have led to changes in the effective components of medicinal materials, while also causing dual residual hazards to both the medicinal materials and the cultivation environment, ultimately posing serious safety risks to human health [25,26]. For instance, Chen et al. observed that the application of CCC increased the yield of *Codonopsis pilosula* by 36%, while the content of lobetyolin decreased by 20% [27]. Subsequently, Liao et al. discovered that CCC could increase the content of amino acids and fatty acids in *Codonopsis pilosula* but reduce the content of poly-saccharides and some volatile substances [28]. In the cultivation of *Scutellaria baicalensis*, CCC significantly reduced the flavonoid content in its underground parts [29]. During the vigorous growth period of *Ginkgo biloba*, the application of CCC significantly increased the content of ginkgolides A and B, bilobalide, and total terpene lactones compared with the control [30].

The application history of CCC in the cultivation of *Angelica sinens* is exceeds 30 years [31]. As a key means to increase yield and improve quality, its effectiveness lies in efficiently curbing the phenomenon of early bolting [32,33,34]. Research clearly indicates that early bolting weakens the biosynthesis process of ferulic acid and flavonoids in ASR and accelerates the lignification process of the roots [35]. Intervention with CCC significantly reduces the incidence of bolting, alleviates the problem of root lignification, and promotes an increase in yield of up to 31% [36]. However, further in-depth exploration and verification are needed regarding the impact of CCC on the overall spectrum of medicinal efficacy components in ASR.

In this research, a comprehensive investigation was carried out on the residue of CCC and its impact on the yield and the active constituents of ASR. Firstly, after spraying CCC onto *Angelica sinensis*, the yield of ASR and the residue of CCC within it were determined. Furthermore, qualitative assessments were conducted using UPLC-QTOF-MS to analyze the influence of CCC at varying concentrations on the overall chemical composition profile of ASR. Subsequently, the chemical composition of ASR was determined, and the levels of nine key active components, comprising two organic acids (ferulic acid and coniferyl ferulate) and seven phthalide compounds (Z-ligustilide, butylphthalide, butylidenephthalide, levistilide A, senkyunolide A, senkyunolide H, and senkyunolide I), were quantitatively assessed using UFLC-MS/MS. This study enhances our comprehension of the impact of different CCC dosages on the yield and quality of ASR, offering a robust scientific backing for the application of CCC in its cultivation.

## 2. Results and Discussion

### 2.1. Effects of CCC on the Yield of ASR

**Figure 1:**
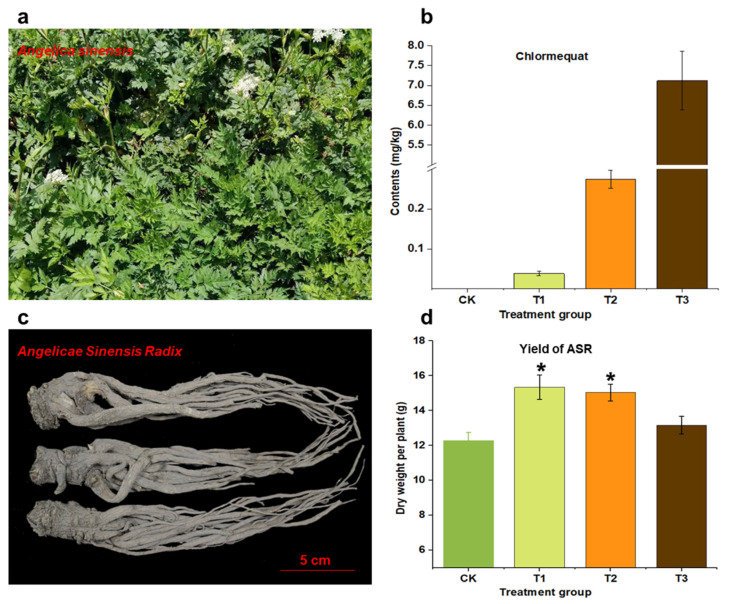
The plant morphology aerial part of Angelica sinensis (**a**); Angelica sinensis radix (**b**); CCC residue in ASR (**c**); and the yield of ASR (**d**). The asterisk represents a statistically significant difference (*p* < 0.05). CK denotes the control group, while T1, T2, and T3 represent CCC concentrations of 0.1 g/L, 1.0 g/L, and 10.0 g/L, respectively.

Plant growth retardants such as CCC are widely used in the production of medicinal plants. By inhibiting the above-ground growth of plants and promoting the growth of underground rhizomes, these regulators can increase the yield of root medicinal materials. The results of this study suggest that the application of CCC enhanced the growth of ASR, as evidenced in Figure 1 and Table S1 (Supplementary Materials). Specifically, when compared with the control group, the single plant root dry weights for treatments T1 (CCC at 0.1 g/L) and T2 (CCC at 1.0 g/L) exhibited significant increases of 25% and 22%, respectively. However, there was no significant change in the single plant root dry weight for T3 (CCC at 10.0 g/L). This suggests that using a higher concentration (10.0 g/L) of CCC does not improve the yield of ASR, but concentrations between 0.1 and 1.0 g/L are beneficial for increasing yield. Our research further confirms the role of CCC in enhancing medicinal material yield. However, our results indicate an increase of only up to 25%, which still shows some discrepancy compared to previous reports. A previous report found intervention with CCC significantly reduces the incidence of bolting, alleviates the problem of root lignification, and promotes an increase in yield of up to 31% [36]. Our results may be influenced by other comprehensive factors during the cultivation process.

### 2.2. Residue of CCC in ASR

We referred to previously established methods when analyzing the residues of CCC in ASR using LC-MS/MS [37]. The results showed that residues were detected in all samples after CCC spraying, and the residue levels were positively correlated with the application concentrations (Figure 1 and Supplementary Table S2). Specifically, at a concentration of 0.1 g/L, the residue of CCC in ASR was approximately 0.04 mg/kg. At concentrations of 1.0 g/L and 10.0 g/L, the residue levels exceeded 0.38 mg/kg and 7.12 mg/kg, respectively. This result is similar to what we previously measured in Astragali Radix, where, at high concentrations, the residue levels of CCC exceeded the maximum residue limits (MRLs) established for relevant foods [38]. Although there is currently no explicit standard for the MRLs of CCC in TCM, the national food safety guidelines specify MRLs for CCC in certain foods. Among the listed food categories, the MRL for oats is 10.0 mg/kg, while the MRLs for other foods are maintained below 5.0 mg/kg, specifically 5 mg/kg for wheat, corn, and canola oil, and below 5.0 mg/kg for other crops such as barley and peanuts. Additionally, the United States, the European Union, Japan, and others have set the MRLs of CCC in foods.

CCC residues are detectable in a variety of crops and can also enter the food chain through livestock feed, leading to their presence in numerous meat and dairy products [39,40]. Additionally, growing evidence suggests that CCC exhibits reproductive toxicity, adversely affecting the growth and development of offspring [41,42]. A particular study found that exposure to CCC below the acceptable daily intake (ADI) level (0.024 mg/kg bw) resulted in decreased fertilization rates in male mice [43]. In 2019, the National Institute for Occupational Safety and Health (NIOSH) identified CCC as a potential endocrine disruptor [44]. The findings of this study clearly indicate that the residue levels of CCC in ASR are significant. Given that ASR is utilized as both a TCM and a food source, the presence of CCC residues could pose health risks to consumers. Therefore, it is essential to evaluate safety concerns regarding acceptable residue levels when employing CCC in the cultivation of *Angelica sinensis*.

### 2.3. Chemical Composition Changes in ASR

**Figure 2:**
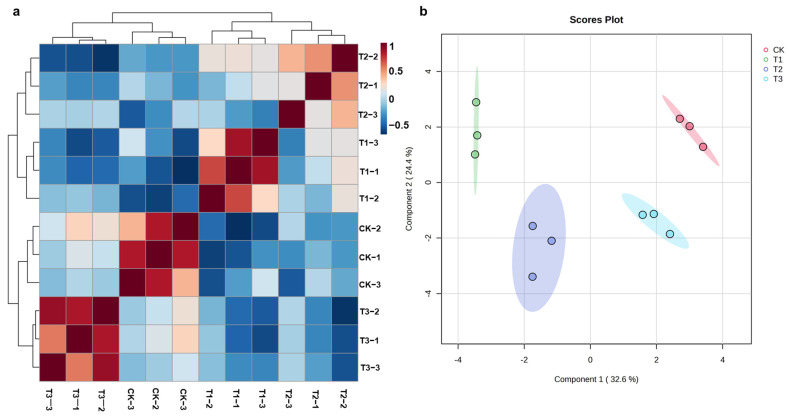
Correlation heatmap (**a**) and scores plot of sPLS-DA (**b**) for different group samples. Red represents a higher correlation; blue represents a negative correlation.

This study used the UPLC-QTOF-MS platform to collect metabolomics data in positive ion mode. The raw data were processed by Progenesis QI v2.0 software, with steps including importing raw data, peak alignment, peak extraction, and normalization, resulting in a peak table with retention time, *m*/*z*, and peak intensity (Supplementary Table S3). The chromatographic peak extraction time range was 1–25 min. The peak table was then used for statistical analysis through the online software MetaboAnalyst 6.0 (https://www.metaboanalyst.ca/, accessed on 30 August 2024). To reflect the differences between samples from each group comprehensively, the original data was scaled using Pareto scaling via MetaboAnalyst 6.0, followed by automatic correlation analysis and sparse partial least squares discriminant analysis (sPLS-DA) between different groups (Figure 2). The results indicated significant differences in chemical components between the control group and CCC-treated groups, with good intra-group correlations, suggesting minimal differences within each sample group. The significant differences between groups may indicate changes in the chemical component profiles of ASR, making it necessary to delve deeper into the analysis of each group.

Currently, it is believed that differences in the efficacy of TCMs are primarily caused by variations in their effective components. However, the efficacy of the same TCM often exhibits diversity, which is reflected in its composition as multiple targets, meaning the effectiveness of the medicine is the result of the combined action of various active components. ASR is a typical TCM with complex active components. Current research identifies phthalides, organic acids, and polysaccharides as its main active constituents. This study examined the alterations in the chemical profile of ASR following the application of CCC from a metabolomics perspective. The results indicated that the use of CCC during the cultivation of *Angelica sinensis* significantly altered the chemical profile of ASR. However, whether these changes directly affect the pharmacological activity or clinical efficacy of ASR requires further experimental research.

To understand the specific chemical components in ASR, we derived and identified the chemical components based on UPLC-QTOF-MS data. We reviewed the relevant literature on the mass spectrometry of ASR compounds and established a mass spectrometry database for ASR. According to the MS1 information of the ASR samples, the feature ions of various components are mainly [M+H]^+^. We performed MS2 fragmentation information analysis using characteristic precursor ions. Based on the MS2 fragmentation information and established database, we identified the compounds in ASR. Ultimately, we preliminarily identified four organic acids, 13 phthalates, and four additional compounds in ASR, with the identification results shown in Table 1 [45,46].

Phthalate compounds are an important class of active ingredients in ASR. Modern research has found that they have a wide range of biological activities, including analgesic, anti-tumor, and neuroprotective effects [47]. Statistics show that a total of 46 phthalate compounds have been identified from ASR, including seven simple phthalates, 21 hydroxy phthalates, and 18 dimeric phthalates. Z-ligustilide is the main active component of the essential oil from ASR, representing the highest proportion [48]. The organic acids in ASR primarily include ferulic acid, succinic acid, vanillic acid, chlorogenic acid, anethole acid, and palmitic acid, among which ferulic acid is the key component used for the quality control of ASR in current pharmacopoeias and is also the first organic acid isolated from ASR. The pharmacological value of the organic acids in ASR is mainly reflected in ferulic acid, whose biological activities include antioxidant effects, the protection of endothelial cells, anti-inflammatory properties, antifibrotic effects, anti-apoptotic effects, and the inhibition of platelet aggregation [9,49]. These components have consistently been the focus of research on *Angelica sinensis*.

### 2.4. Quantitative Analysis of Nine Bioactive Compounds in ASR

#### 2.4.1. Optimization of the HPLC-MS/MS Condition

In order to establish the optimal analytical conditions for the target compounds in ASR, we conducted a systematic optimization of both the chromatographic conditions and mass spectrometry parameters. The composition of the mobile phase has a significant impact on ionization efficiency and the separation of analytes. Drawing upon previous studies [2,50], we identified the most appropriate mobile phase composition and chromatographic conditions to guarantee optimal peak shape, response intensity, and resolution. Comprehensive details regarding the optimal chromatographic conditions are provided in the Section 3.

**Figure 3:**
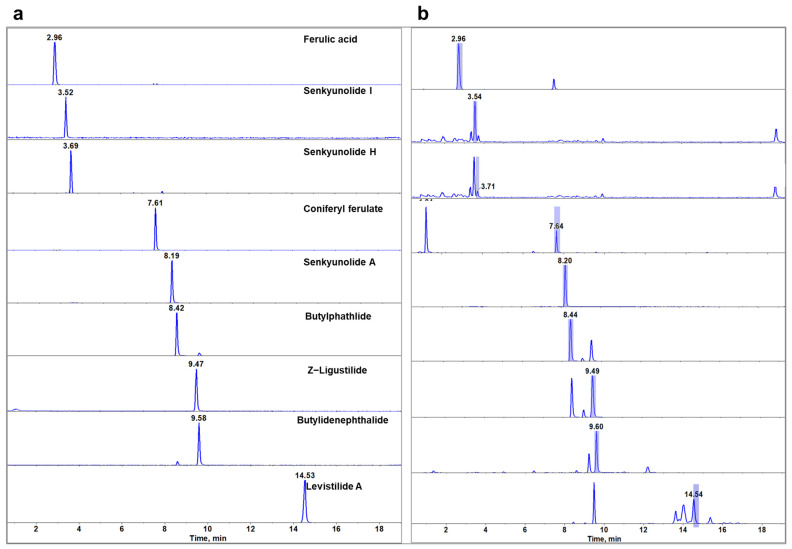
Extracted ion chromatograms (EIC) of analytes from the mixed standard (**a**) and ASR (**b**).

To determine the optimal mass spectrometric behavior and parameters for the identification and quantification of target analytes, a syringe pump (Harvard Apparatus, South Natick, MA, USA) was used to introduce standard solutions of each analyte (100 ng/mL) directly into the mass spectrometer at a flow rate of 10 μL/min. The critical mass spectrometry parameters, namely, declustering potential (DP) and collision energy (CE), were meticulously fine-tuned to achieve optimal sensitivity. The quantitative method employed the multiple reaction monitoring (MRM) scan mode. The optimized MRM transitions and their parameters are shown in Table 2, while the representative chromatogram of the target analytes is depicted in Figure 3.

#### 2.4.2. Sample Pretreatment

To obtain the optimal extraction conditions for the target analytes, we optimized various factors, including the extraction solvents (methanol, ethanol, and acetonitrile), solvent concentrations (50%, 70%, and 100%, *v*/*v*), solvent volumes (10, 20, and 30 mL), and extraction times (15, 30, 45 min, and 1 h). By comparing the chromatographic peaks of different components under various extraction conditions, we ultimately determined the best extraction conditions for the effective components of ASR to be 0.5 g of sample, 30 mL of 70% methanol, and an ultrasonic extraction time of 30 min. Under these conditions, the recovery rates for three concentrations (20.0 ng/L, 50.0 ng/L, and 100 ng/L) fell within an acceptable range (82.6–97.9%), with the specific results shown in Table 3.

#### 2.4.3. Method Validation

The results of our methodological experiments show that nine target components exhibited good linear relationships within the corresponding concentration ranges, with correlation coefficients (*r*) all exceeding 0.9992 (Table 3). The limit of detection and limit of quantification for all analytes were below 10 ng/mL and 5 ng/mL, respectively. The intra-day precision RSD values for the measured components ranged from 2.16 to 5.55%, while the inter-day precision RSD values ranged from 1.15% to 5.72%. The recovery rates for the nine analytes were between 81.88% and 112.86%. The samples were measured every 12 h over a 48-h period, and the RSD of the peak areas for all measured components was less than 5.81%. The repeatability RSD values for the measured components ranged from 1.69 to 5.01%. These results indicate that the established method is simple, rapid, highly sensitive, stable, and reliable, meeting the requirements for the determination of the active components in ASR.

#### 2.4.4. Sample Analysis

**Figure 4:**
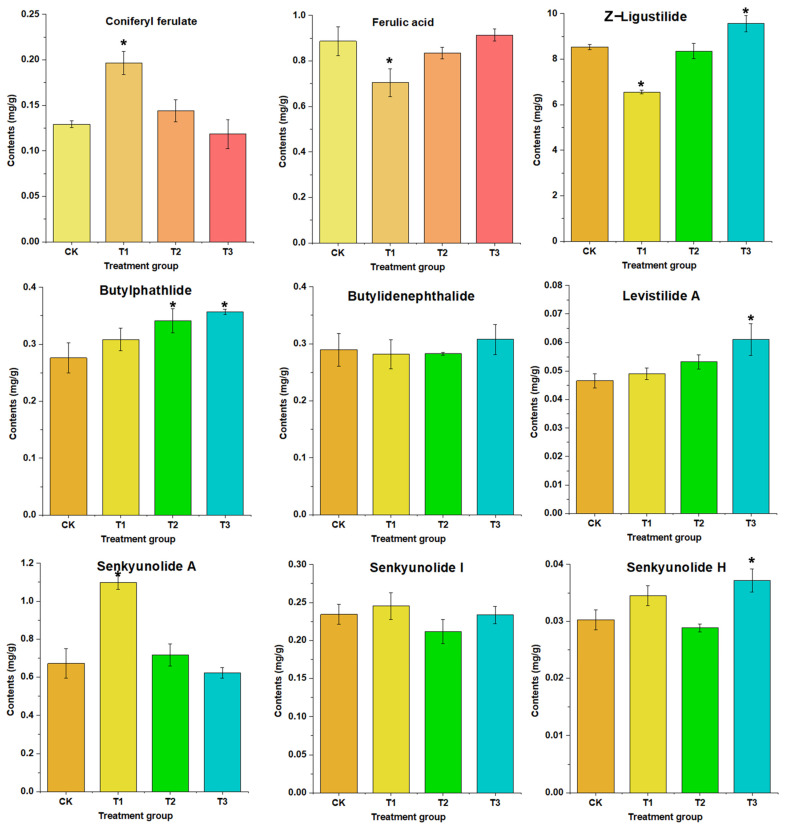
The contents of nine compounds in ASR from different groups. The asterisk represents a statistically significant difference (*p* < 0.05). Nine target compounds including coniferyl ferulate, ferulic acid, ethyl ferulate, levistilide A, Z-ligustilide, butyldenphthalide, senkyunolide I, senkyunolide A, columbianadin, butylphathlide, and senkyunolide H.

The above method was used to detect nine target components in 18 samples of ASR. The identification of the target components was performed based on retention times and characteristic ion pairs, and a quantitative analysis was conducted using the external standard method. The quantitative experimental results indicate (Table 4, Figure 4) that among all target analytes, Z-ligustilide had the highest content, followed by senkyunolide A and ferulic acid. The application of different concentrations of CCC promoted an increase in the contents of butylphathlide and levistilide A in ASR, with a more significant effect observed as the concentration increased. Compared with the control group, at a concentration of 10.0 g/L, butylphathlide and levistilide A increased by 15.93% and 24.63%, respectively. The application of CCC at a low concentration of 0.1 g/L significantly increased the contents of coniferyl ferulate and senkyunolide A in ASR, with increases of 51.97% and 63.62% compared with the control. However, at higher concentrations (1.0 g/L and 10.0 g/L), no significant effects were observed, and a decreasing trend was noted instead. Additionally, at low concentrations (0.1 g/L), CCC significantly reduced the contents of Z-ligustilide and ferulic acid in ASR, with decreases of 20.53% and 23.15%, respectively. As the application concentration increased, this downregulation effect weakened, and at high concentrations (10.0 g/L), the content of Z-ligustilide was significantly higher than that of the control, showing an inversion trend. Overall, the results indicate that CCC exhibits a complex regulatory trend on multiple active components in ASR, with different concentrations showing varying regulatory effects on the same component and the same concentration displaying different regulatory trends on different components. These changes in component levels affect the overall proportion of active ingredients in ASR.

Modern pharmacological studies have shown that phthalide components have multiple effects, including inhibiting platelet aggregation, anti-thrombus formation, anti-tumor activity, neuroprotection, analgesia, and anti-shock properties [47]. The main organic acids include ferulic acid and coniferyl ferulate. These components exhibit effects such as anti-platelet aggregation, vasodilation, and antioxidant activity [51]. Because of the varying trends in different active components, changes in their proportions in ASR extracts are inevitable, which can alter the efficacy of related drugs containing ASR and potentially lead to unexpected clinical outcomes. Therefore, in further in-depth studies, the use of CCC in *Angelica sinensis* should be approached with caution. While increasing the yield of medicinal materials, it is also essential to focus on the changing trends in active components.

## 3. Materials and Methods

### 3.1. Chemicals and Reagents

A 50% aqueous solution of CCC was obtained from Sichuan Guoguang Agrochemical Co., Ltd. (Chengdu, China), while the CCC standard compound, possessing a purity of 98.0%, was sourced from the Agro-Environmental Protection Institute (Tianjin, China). Standard compounds with purities exceeding 98.0%, confirmed by HPLC-UV analysis, included coniferyl ferulate, ferulic acid, ethyl ferulate, levistilide A, Z-ligustilide, butyldenphthalide, senkyunolide I, senkyunolide A, columbianadin, butylphathlide, and senkyunolide H, all provided by Chengdu Must Biotechnology Co., Ltd. (Chengdu, China). HPLC-grade acetonitrile and methanol were obtained from Fisher Scientific (Fair Lawn, NJ, USA). Additional reagents and chemicals were purchased from Sinopharm Chemical Regent Beijing Co., Ltd. (Beijing, China). The ultrapure water utilized for HPLC-MS/MS analysis was generated using a Milli-Q system manufactured by Merck Millipore (Billerica, MA, USA).

### 3.2. Plant Materials and CCC Treatment

Biennial *Angelica sinensis* plants were grown at an experimental site in Sigou Town, Minxian County, Gansu Province. The morphology of the aerial parts and rhizomes is depicted in Figure 1. The planting density was approximately 10~15 plants per square meter, with the area divided into multiple experimental plots of about 30 m^2^ each. A 50% CCC aqueous solution was diluted to concentrations of 10.0, 1.0, and 0.1 g/L. These solutions were uniformly sprayed onto the leaves using small sprayers. Each concentration was tested in three randomly chosen plots, with a second application conducted one week after the initial application. ASR harvesting occurred four months after CCC treatment, while the control group received only water. Post-harvest, the ASR samples were dried, and the number of plants and weight of ASR were recorded to determine the average yield per plant. The dried samples were then crushed, homogenized, and stored in Ziplock bags at 4 °C until analysis. The identification of *Angelica sinensis* was verified by Professor Xiaojun Ma, and voucher specimens (VS-P-2022-26 for the plant and VS-S-2022-671 for the samples) were deposited at the Institute of Medicinal Plant Development, Chinese Academy of Medical Sciences, and Peking Union Medical College in Beijing, China.

### 3.3. Instruments

Compound identification was performed using an Acquity UPLC H-Class system paired with an Xevo G2-S QTof™ mass spectrometer (Waters, Milford, MA, USA). For quantitative analysis, an Agilent 1260 series HPLC system (Agilent Technologies, Palo Alto, CA, USA) was coupled with a QTRAP^®^ 4500 mass spectrometer (AB SCIEX, Foster City, CA, USA). The preparation of samples and mobile phases involved the use of various types of equipment, including a KQ-400DE ultrasonic cleaning machine manufactured by Kunshan Ultrasonic Instrument Co., Ltd. (Kunshan, China), a Milli-Q purification system from Millipore (Bedford, MA, USA), a GZX-9070MBE electric blast drying oven supplied by Shanghai Boxun Industrial Co., Ltd. (Shanghai, China), and electronic analytical balances (BT25S 1/100,000 and BS210S 1/10,000) sourced from Sartorius Scientific Instrument (Beijing) Co., Ltd. (Beijing, China).

### 3.4. Standard and Sample Solution Preparation

Stock solutions of coniferyl ferulate, ferulic acid, ethyl ferulate, levistilide A, Z-ligustilide, butyldenphthalide, senkyunolide I, senkyunolide A, columbianadin, butylphathlide, and senkyunolide H were individually prepared at a concentration of 0.1 mg/mL in methanol. These stock solutions of standards were subsequently diluted using a mixed solvent of methanol and water (70:30, *v*/*v*) to create mixed standard working solutions. All prepared solutions were stored in a refrigerator at 4 °C until analysis. Six different concentrations of mixed standard were analyzed, and calibration curves were generated by plotting the peak area against the concentration of the target analytes.

For the analysis of CCC residue, 1.0 g of homogenized ASR powder was accurately weighed and placed in a 50 mL centrifuge tube. It was then combined with 10 mL of acetonitrile containing 1% formic acid for extraction. The mixture underwent vortex mixing for 1 min, followed by ultrasonic extraction at 40 kHz for 10 min. After extraction, the samples were centrifuged at 10,000× *g* for 5 min, and the supernatant was diluted one time with ultrapure water. After another minute of vortex mixing, the solution was filtered through a 0.22 μm nylon syringe filter and stored at 4 °C until further analysis.

For the chemical composition analysis of ASR, the homogenized samples (0.5 g) were precisely weighed and subjected to ultrasonic extraction at 40 kHz with 30 mL of a methanol–water mixture (70:30, *v*/*v*) for 30 min. Then, 1.0 mL of the upper layer of the extracted solution was filtered through a 0.22 µm syringe nylon filter before analysis. For the quantitative analysis of nine active compounds in ASR, homogenized samples (0.5 g) were accurately weighed and subjected to ultrasonic extraction at 40 kHz with 30 mL of a methanol–water mixture (70:30, *v*/*v*) for 30 min. Following this, 1.0 mL of the extract was pipetted and diluted to 10 mL using the same solvent. The solution was mixed using a vortex for 30 sec and then filtered through a 0.22 μm nylon syringe filter. All samples were stored in a refrigerator at 4 °C until analysis.

### 3.5. Chemical Composition Analysis of ASR

The compounds in ASR were identified using an Acquity UPLC H-Class system paired with an Xevo G2-S QTof™ mass spectrometer (Waters, Milford, MA, USA). Chromatographic separation utilized an ACQUITY UPLC™ HSS T3 column (100 mm × 2.1 mm, 1.8 μm, Waters) maintained at 35 °C, with a flow rate of 0.3 mL/min. The mobile phase comprised water containing 0.1% formic acid (A) and acetonitrile (B), employing a gradient elution as follows: 95–60% (A) from 0 to 8 min; 60–57% (A) from 8 to 12 min; 57–33% (A) from 12 to 18 min; 33–25% (A) from 18 to 24 min; 25–5% (A) from 24 to 25 min; maintaining at 5% A from 25 to 30 min; 5–95% (A) from 30 to 31 min; and then equilibration at 95% A from 31 to 35 min.

Mass spectrometry with electrospray ionization (ESI) was conducted in positive mode with the following settings: capillary voltage at 3 kV, sampling cone voltage at 30 V, extraction cone voltage at 4.0 V, source temperature maintained at 100 °C, and desolvation temperature set at 300 °C. A collision energy of 15 eV was utilized during the MS acquisition, and 45 eV was utilized for the MSE acquisition. The cone gas flow rate was maintained at 50 L/h. Time-of-flight (TOF) MS scanning covered a mass range of *m*/*z* 100–1200 Da. Data were collected using MassLynx V4.1 software.

### 3.6. Quantitative Analysis of Nine Components in ASR

#### 3.6.1. HPLC-QTRAP-MS/MS Conditions

Quantitative analysis was conducted using an Agilent 1260 series HPLC system (Agilent Technologies, Palo Alto, CA, USA), coupled to a QTRAP^®^ 4500 mass spectrometer (AB SCIEX, Foster City, CA, USA) through an ESI interface. Chromatographic separation was achieved using an Agilent Poroshell 120 EC C18 column (100 mm × 3.0 mm, 2.7 µm; Agilent, Palo Alto, CA, USA). The mobile phase consisted of water with 0.1% formic acid (A) and acetonitrile (B), with the following gradient elution: 80–65% (A) from 0 to 1 min; 65–60% (A) from 1 to 4 min; 60–45% (A) from 4 to 5 min; 45–40% (A) from 5 to 10 min; 40–20% (A) from 10 to 16 min; maintaining at 10% A from 16 to 17 min; and then equilibration at 80% A from 17 to 20 min. The column was operated at a flow rate of 0.3 mL/min, with an injection volume of 5.0 μL.

Mass spectrometry detection was performed using an electrospray ionization (ESI) source in both positive and negative modes. The curtain gas (CUR), nebulizer gas (GS1), and auxiliary gas (GS2) were set at 35, 50, and 50 psi, respectively. The ion spray voltage (IS) was adjusted to 5500 V for positive mode and −4500 V for negative mode, and the source temperature was maintained at 450 °C. Multiple reaction monitoring (MRM) mode was utilized for quantitation, with a dwell time of 50 ms for each MRM transition. The UFLC-QTRAP-MS/MS system was controlled, and data acquisition and processing were handled using Applied Biosystems Analyst software (version 1.6).

#### 3.6.2. Method Validation

In accordance with the International Conference on Harmonization (ICH) guidelines for analytical method validation [52], the method was assessed by linearity, sensitivity, precision (both intra-day and inter-day), stability, repeatability, and accuracy. Calibration curves were generated by plotting the analyte concentration (X) against the peak area (Y), with at least six concentration levels analyzed in triplicate. Limits of detection (LOD) and quantification (LOQ) were established using signal-to-noise (S/N) ratios of approximately 3 and 10, respectively. Precision was evaluated through repeated analyses (*n* = 6) of standard samples within one day (intra-day) and across three consecutive days (inter-day). Accuracy was tested by spiking target analytes at three different concentrations into previously analyzed samples. Stability was evaluated by analyzing the sample solution at room temperature, with assessments conducted every 12 h over a period of 2 days. For repeatability, six independent sample solutions were prepared and investigated according to sample preparation method of quantitative analysis.

## 4. Conclusions

During the cultivation of *Angelica sinensis*, the application of CCC at appropriate concentrations can enhance the yield of ASR. However, our chemical composition analysis show that varying concentrations of CCC significantly alter the chemical profile of ASR, resulting in changes to its quality as a medicinal herb. Our quantitative analyses indicate that CCC exerts a complex regulatory effect on the active components of ASR. High concentrations of CCC promote the accumulation of butylphthalide and levistilide A; however, they also lead to significant residues of CCC in ASR. Conversely, low concentrations of CCC not only increase the yield of ASR but also boost the accumulation of coniferyl ferulate and senkyunolide A, albeit leading to a notable decrease in the levels of crucial active constituents such as Z-ligustilide and ferulic acid within ASR. This indicates that CCC has a significant regulatory effect on the active components of ASR, leading to changes in the proportions of various active compounds. Given the residual presence of CCC in ASR and its intricate regulatory effects, achieving a balance between yield and quality presents a challenge. Therefore, it is important to apply CCC cautiously in *Angelica sinensis* cultivation to ensure the quality and safety of ASR.

## Figures and Tables

**Table 1:** The detailed information of the identified compounds in ASR.

Compound No.	Rt (min)	Formula	Expected *m*/*z*	Measured *m*/*z*	Mass Error (ppm)	Fragment Ion	Compound	Classification
1	3.121	C_11_H_12_N_2_O_2_	205.0977	205.0978	0.49	188.0700, 170.0600	Tryptophan	amino acid
2	3.603	C_16_H_18_O_9_	355.1029	355.1028	−0.36	163.0392, 145.0300, 135.0439	Chlorogenic acid	organic acids
3	5.623	C_10_H_10_O_4_	195.0657	195.0645	−6.15	177.0545, 137.9867	Ferulic acid	organic acids
4	5.833	C_12_H_14_O_4_	223.097	223.0605	−163.39	209.1170, 177.0518	Ethyl ferulate	organic acids
5	6.944	C_12_H_16_O_4_	225.1126	225.1141	6.66	207.1011, 189.0927	Senkyunolide I	phthalides
6	7.269	C_12_H_16_O_4_	225.1126	225.1141	6.66	207.1011, 189.0927	Senkyunolide H	phthalides
7	8.808	C_12_H_12_O_2_	189.0915	189.0917	1.07	171.0733, 161.0971, 143.0789	Butylidenephthalide isomer	phthalides
8	11.230	C_16_H_20_O_9_	357.1185	357.1206	5.96	177.0552	Coniferyl ferulate	organic acids
9	14.139	C_12_H_16_O_2_	193.1228	193.1230	0.98	175.1132, 147.1171, 137.0605	Senkyunolide A	phthalides
10	14.609	C_12_H_14_O_2_	191.1072	191.1080	4.31	173.0965, 145.1014	Butylphathlide isomer	phthalides
11	14.667	C_18_H_37_NO_3_	316.2851	316.2852	0.43	145.1014	Dehydrophytosphingosine	fatty acids
12	15.587	C_12_H_14_O_2_	191.1072	191.1074	1.31	173.0965, 145.1014	Butylphathlide	phthalides
13	16.344	C_12_H_14_O_2_	191.1072	191.1089	8.64	173.0965, 145.1014	Z-ligustilide	phthalides
14	17.661	C_12_H_14_O_2_	191.1072	191.1069	−1.57	173.0965, 145.1014	Ligustilide isomer	phthalides
15	18.437	C_24_H_40_O_6_	425.2903	425.2900	−0.60	345.4098, 121.1012	1-beta-Hydroxycholic acid	fatty acids
16	19.147	C_12_H_12_O_2_	189.0915	189.0920	2.43	171.0733, 161.0971, 143.0789	Butylidenephthalide	phthalides
17	20.667	C_24_H_28_O_4_	381.2065	381.2065	−0.09	191.1069, 149.0592	Levistilide A isomer	phthalides
18	20.847	C_24_H_28_O_4_	381.2065	381.2065	−0.09	191.1069, 149.0592	Levistilide A isomer	phthalides
19	21.343	C_24_H_28_O_4_	381.2065	381.2064	−0.21	191.1069, 149.0592	Levistilide A	phthalides
20	23.307	C_19_H_20_O_5_	329.1389	329.1397	2.51	191.1069, 174.1264	Columbianadin	phthalides
21	27.371	C_18_H_37_ON	284.2953	284.2942	−3.82	174.1291	Stearamide	fatty acids

**Table 2:** The optimized MS/MS parameters for the targeted analytes.

Compound	Rt (min)	Precursor Ion (*m*/*z*)	Product Ions (*m*/*z*)		Ionization Mode	DP (V)	CE (V)
			For Quantification	For Identification			
Ferulic acid	2.95	192.9	133.8	177.7	ESI−	−80	−15/20
Senkyunolide I	3.51	225.2	207.2	161.1	ESI+	70	15/30
Senkyunolide H	3.7	225.2	207.2	161.1	ESI+	70	15/30
Coniferyl ferulate	7.61	355.3	192.7	133.7	ESI−	−70	−15/30
Senkyunolide A	8.18	193	137	146.8	ESI+	20	20/15
Butylphathlide	8.41	190.9	144.9	173	ESI+	20	15/15
Z-ligustilide	9.46	190.9	173	144.9	ESI+	20	15/15
Butylidenephthalide	9.56	189.2	170.9	128	ESI+	80	20/25
Levistilide A	14.52	381.3	191	157.2	ESI+	20	25/35

**Table 3:** Method validation results including the linearity, limit of detection (LOD), limit of quantification (LOQ), precision, recovery, repeatability, and stability.

Compound	Rt (min)	Linearity		LOD (ng/mL)	LOQ (ng/mL)	Precision (RSD, %)		Recovery (%)			Repeatability (RSD, %)	Stability (RSD, %)
		Range (ng/mL)	r			Intra-Day	Inter-Day	Low Level	Medium Level	High Level		
Ferulic acid	2.95	2–500	0.9997	0.6	2	2.16	1.15	105.02	112.86	109.29	2.34	3.66
Senkyunolide I	3.51	10–500	0.9992	5	10	2.42	4.37	101.39	109.46	110.01	4.31	5.81
Senkyunolide H	3.7	10–500	0.9998	5	10	2.63	5.56	105.26	111.07	112.33	2.04	2.66
Coniferyl ferulate	7.61	5–500	0.9992	0.6	2	3.22	5.72	100.24	100.91	102.74	5.01	4.05
Senkyunolide A	8.18	2–500	0.9996	0.6	2	4.20	2.85	82.35	83.18	85.14	1.69	1.89
Butylphathlide	8.41	2–100	0.9993	0.6	2	3.89	3.10	85.06	83.34	81.88	2.48	1.67
Z-ligustilide	9.46	15–500	0.9999	2	5	5.55	3.36	90.55	89.37	93.01	1.99	2.68
Butylidenephthalide	9.56	2–500	0.9998	0.6	2	3.74	4.52	94.22	87.05	89.76	4.35	2.15
Levistilide A	14.52	2–100	0.9992	0.6	2	4.49	2.90	91.17	90.19	90.03	2.29	2.38

**Table 4:** The contents of nine compounds in ASR (mg/g, *n* = 3).

Compounds	Treatment Groups			
	CK	T1	T2	T3
Ferulic acid	0.8867	0.7046	0.8352	0.9142
Senkyunolide I	0.2342	0.2453	0.2116	0.2335
Senkyunolide H	0.0302	0.0345	0.0288	0.0372
Coniferyl ferulate	0.1293	0.1965	0.1441	0.1185
Senkyunolide A	0.6714	1.0987	0.7171	0.6224
Butylphathlide	0.2763	0.3081	0.3413	0.3572
Z-ligustilide	8.5304	6.5549	8.3521	9.5662
Butylidenephthalide	0.2898	0.2820	0.2827	0.3076
Levistilide A	0.0466	0.0490	0.0532	0.0610

## Data Availability

Data are contained within the article and Supplementary Materials.

## Supplementary Materials

The following supporting information can be downloaded at https://www.mdpi.com/article/10.3390/molecules29194725/s1: Table S1: The yield of ASR after CCC treatment; Table S2: CCC residue levels in different treatment groups of ASR; Table S3: The metabolites peak table obtained by UPLC-QTOF-MS.
